# The nutritional quality of foods carrying health-related claims in Germany, The Netherlands, Spain, Slovenia and the United Kingdom

**DOI:** 10.1038/ejcn.2016.114

**Published:** 2016-07-13

**Authors:** A Kaur, P Scarborough, S Hieke, A Kusar, I Pravst, M Raats, M Rayner

**Affiliations:** 1 British Heart Foundation Centre on Population Approaches for Non-Communicable Disease Prevention, Nuffield Department of Population Health, University of Oxford, Oxford, UK; 2 European Food Information Council (EUFIC), Brussels, Belgium; 3 Nutrition Institute, Ljubljana, Slovenia; 4 Biotechnical Faculty, University of Ljubljana, Ljubljana, Slovenia; 5 Food, Consumer Behaviour and Health Research Centre, University of Surrey, Surrey, UK

## Abstract

**Backgroung/Objectives:**

Compares the nutritional quality of pre-packaged foods carrying health-related claims with foods that do not carry health-related claims.

**Subject/Methods:**

Cross-sectional survey of pre-packaged foods available in Germany, The Netherlands, Spain, Slovenia and the United Kingdom in 2013. A total of 2034 foods were randomly sampled from three food store types (a supermarket, a neighbourhood store and a discounter). Nutritional information was taken from nutrient declarations present on food labels and assessed through a comparison of mean levels, regression analyses and the application of a nutrient profile model currently used to regulate health claims in Australia and New Zealand (Food Standards Australia New Zealand's Nutrient Profiling Scoring Criterion, FSANZ NPSC).

**Results:**

Foods carrying health claims had, on average, lower levels, per 100 g, of the following nutrients, energy—29.3 kcal (*P*<0.05), protein—1.2 g (*P*<0.01), total sugars—3.1 g (*P*<0.05), saturated fat—2.4 g (*P*<0.001), and sodium—842 mg (*P*<0.001), and higher levels of fibre—0.8 g (*P*<0.001). A similar pattern was observed for foods carrying nutrition claims. Forty-three percent (confidence interval (CI) 41%, 45%) of foods passed the FSANZ NPSC, with foods carrying health claims more likely to pass (70%, CI 64%, 76%) than foods carrying nutrition claims (61%, CI 57%, 66%) or foods that did not carry either type of claim (36%, CI 34%, 38%).

**Conclusions:**

Foods carrying health-related claims have marginally better nutrition profiles than those that do not carry claims; these differences would be increased if the FSANZ NPSC was used to regulate health-related claims. It is unclear whether these relatively small differences have significant impacts on health.

## Introduction

Diet is a leading risk factor for non-communicable disease in Europe,^1^ and 90% of deaths in the European Union (EU) are due to non-communicable diseases.^2^ Therefore, improving diet should be a public health goal as even small improvements can have large population benefits.^3, 4^ The World Health Organization recommends that, as part of a healthy diet, adults should consume at least five portions of fruit and vegetables a day. The World Health Organization also recommends limiting fat intake to <30% of the total energy intake, saturated fat to <10% of the total energy intake, and free sugars to 5–10% of the total energy intake and to consume <5 g of salt per day.^5^

Within the EU, the laws regarding health-related claims are set out in the 1924/2006 Regulation on nutrition and health claims for food.^6^ In the Regulation, a health claim is defined as ‘any claim that states, suggests or implies that a relationship exists between a food category, a food or one of its constituents and health',^7^ whereas nutrition claims are 'any claim that states, suggests or implies that a food has particular beneficial nutritional properties due to the energy, nutrients or other substances it contains, contains in reduced or increased proportions or does not contain'.^6^

In the EU, manufacturers may only use a specific nutrition or health claim if it has been listed in the EU register of nutrition and health claims^7, 8^ and meets the corresponding conditions. If a manufacturer wishes to use a new health claim on any food packaging or marketing materials on the market in Europe, the claim must first be authorised by the European Commission (EC). In order for a health claim to be authorised, manufacturers must submit a dossier containing evidence of the relationship described in the claim, which is then assessed by the European Food Safety Authority. After such a verification procedure, the claim is authorised by the EC through the Comitology procedure.^8^ In addition to being scientifically accurate, the regulation^6^ stipulates that health claims must also be ‘truthful, clear, reliable and useful to the consumer in choosing a healthy diet' (p.7). There are additional stipulations regarding the use of health-related claims outlined in the 1924/2006 Regulation,^6^ and all foods carrying a health or a nutrition claim must provide nutrient declarations.^9^ Studies from EU countries estimate that 7–14% of prepacked food is labelled with health claims or symbols.^10, 11^

Health-related claims may help consumers identify healthier foods by providing useful information to consumers about healthier choices.^12, 13, 14^ On the other hand, research has suggested that health-related claims might be of negligible assistance or might even hinder consumers in their decision-making for a variety of reasons^15, 16, 17^ including neglecting other, more useful sources of information.^18^ However, little is known about the effects of claims on consumer understanding, purchasing and consumption of foods, particularly in real-life shopping situations. The pan-European research project Role of health-related CLaims and sYMBOLs in consumer behaviour (CLYMBOL) has set out to address this lack of knowledge, for an overview of the project see Hieke *et al.*^19^

Some consumers may perceive foods carrying health-related claims more positively because of the presence of a claim (positivity bias).^20^ Despite contention around this area, it remains important to assess the nutritional composition of foods carrying health-related claims. Within the EU, there have been few studies that assess whether foods carrying health-related claims have a better nutritional composition than foods that do not. A recent survey of health and nutrition claims in the United Kingdom found that foods carrying health claims were, on average, slightly healthier than foods that do not carry such claims.^21^ Similar results have also been observed internationally. A survey of health symbols in Canada found few nutritional differences between foods carrying health symbols and those that do not.^22^ Conversely, a survey in North Dakota revealed that 49% of foods carried a health-related claim, and of these 48% had ⩾20% saturated fat, sodium and/or sugar. This increased to 73% when examining the nutrient levels of foods carrying nutrient content claims but was much lower (9%) for health claims.^23^ However, these studies all sampled foods differently, for example, randomly selecting foods through a retailer's website,^21^ sampling all foods within multiple stores in a single city^23^ or sampling foods from the largest retailers,^22^ making comparisons between studies problematic. This study involved sampling products from a number of European countries in order to investigate inter-country differences in the use of claims on food products, on a comparable basis. In this paper, we compare the mean levels of energy, protein, carbohydrate, total sugars, total fat, saturated fat, fibre and sodium for foods bearing health-related claims against those that do not. Although assessing individual nutrients is useful, looking at each nutrient in isolation may not reveal whether the presence of a health-related claim is ‘masking' a poor overall nutrient quality. To address this issue, a nutrient profile model was scheduled to be introduced in the EU in 2009, but this part of the legislation has not yet been implemented. The EC recently announced that it will evaluate whether nutrient profile models are necessary for the implementation of the health claims regulation.^24^ Nutrient profiling is ‘the science of classifying or ranking foods according to their nutritional composition for reasons related to preventing disease and promoting health'.^25^ In this study, we apply the Food Standards Australia New Zealand's Nutrient Profiling Scoring Criterion (FSANZ NPSC), which is currently used to regulate health claims,^26^ and compare the proportion of foods with and without claims that ‘pass' the model.

The research questions for this study are as follows:

1. Are foods that carry a health-related claim healthier than those that do not?
2. Does this differ by type of claim? (health claims vs nutrition claims)
3. Are there differences by food category?
4. Are there country differences?

## Materials and methods

### Data collection and sampling

Data were collected as part of the CLYMBOL project; for an overview of the project see Hieke *et al*,^19^ and for a more detailed description of the data collection methods see Hieke *et al.*^11^

Data collection for this study took place in supermarkets, neighbourhood and discount stores in the United Kingdom, Germany, The Netherlands, Spain and Slovenia in August 2013. These countries were chosen on the basis of geographical spread within the EU and the localities of collaborators of the CLYMBOL project. Approximately 400 foods were sampled in each country, of which 250 were sampled from a supermarket (or a national retailer), 75 from a discounter store and the remaining 75 from a neighbourhood store. The study was powered to detect the differences in the prevalence of health claims on foods between countries. A power calculation was conducted with various sample sizes in order to estimate the precision of the results. After taking time and budget constraints into account, a sample size of 400 foods per country was used, which would produce confidence levels of ±5%, and thus a 10% difference in the prevalence of claims between countries could be detected.

A stratified random sampling method was used in which most pre-packaged foods (‘foods' shall refer to foods and drinks unless otherwise specified) available to purchase on the day of sampling were eligible for inclusion. The following groups of foods were excluded from the sampling frame:

1. Non-food items, that is, items included in appropriate food categories but that are not foods—for example, birthday candles under ‘Food Cupboard/Baking'.
2. Unpackaged foods.
3. Alcoholic drinks (including low alcohol drinks).
4. Food supplements.
5. Deli-style products and all additional products within the ‘Fresh Food/Counters' category, as the majority of products are sold unpackaged; a number of potentially eligible products within this category would have been excluded. This was a compromise on grounds of sampling practicality.

The sampling method was piloted and a standard routine was devised, which was followed by local researchers in the five countries. All sampled foods were purchased, and the packaging was retained. The health-related claims were recorded and categorised as described below. Where it was provided, the nutritional information (energy, protein, carbohydrates, total sugars, fat, saturated fat, fibre and sodium) per 100 g, and selected ingredient compositional data (for example, the proportion of fruit and vegetables), was also recorded. The nutritional information was recorded for the food as consumed rather than as packaged (for example, the nutritional information for reconstituted dried soups was recorded); however, this was done in a manner that made the least possible difference to the raw food while still being edible—for example, breakfast cereals were recorded as consumed without milk.

### Categorisation

The FSANZ NPSC evaluates foods by awarding points to foods on the basis of the levels of ‘positive' nutrients (protein, fibre and the proportion of fruit and vegetables) present in a food, and these points are then deducted from points scored for the levels of ‘negative' nutrients (energy, total sugars, saturated fat and sodium). If a food's final score is >0 (or >3 for drinks or >27 for fats, oils and cheeses), then the food fails the model and is thus not permitted to carry a health claim. In order to apply the FSANZ NPSC, the foods were categorised into the three food groups used by the model (beverages; cheese, oils and spreads; other).

For the analyses and presentation of results, foods were categorised using the food groupings used by the UK's Eatwell Guide.^27^ The Eatwell Guide is a graphical representation of the government's advice around which food group consumers should consume more or less. In addition to the five food groups described in the Eatwell Guide documentation, two new categories were created to capture foods that did not fall into the established categories: composite foods—containing foods that consist of two or more food groups—for example, pizza and ready meals—and miscellaneous foods—containing foods that are not captured by the Eatwell Guide such as spices, cooking aids and so on.

All health-related claims, irrespective of their EC approval status, were categorised using the International Network for Food and Obesity/non-communicable disease Research, Monitoring and Action Support taxonomy.^28^ This taxonomy was chosen because of its compatibility with the Codex Alimentarius Commission (Codex) international definitions^29^ and the EU 1924/2006 Regulation.^6^

The following were not considered as health-related claims:^21^

1. The terms ‘natural', ‘organic' and ‘Halal'.
2. Information on the absence of additives, preservatives, colourings and flavourings.
3. Allergy advice (for example, ‘contains nuts').
4. Statements in relation to specific diets, for example, dairy and/or lactose free, wheat and/or gluten free and vegetarian (or vegan).
5. Storage advice (for example, ‘stays fresh for longer').
6. Reference to the presence of a ‘food or food group' in the product that does not state, suggest or imply a health benefit (for example, ‘contains chocolate').
7. Advertising in relation to sport (for example, ‘official product of the Olympics') or to health concerns unrelated, or only loosely related, to a healthier diet (for example, ‘supporting breast cancer research').
8. Nutrition labelling (either back of pack or front of pack), for example, traffic-light labelling for specific nutrient levels.

### Additional data sources

At the time of data collection, provision of nutritional information on food packaging was only mandatory for foods that carry health-related claims. Therefore, there was incomplete nutritional information, limiting the number of foods that could be tested with the FSANZ NPSC. Consequently the UK Nutrient Databank^30^ was used to supplement the data. The UK Nutrient Databank is a food compositional table containing ~8000 generic foods and the average nutritional values for a wide range of nutrients and micro nutrients. Each food sampled in the study was matched with a similar food in the UK Nutrient Databank by a local nutrition researcher in each of the five countries. In order to assess the validity of this matching process, the nutritional information recorded from the food packaging was compared with the nutritional information from the matched product in the UK Nutrient Databank using Pearson's R correlation statistic. The supplemented data were only used for the application of the FSANZ NPSC. A further analysis of the validity of this matching process was conducted on the sample of Slovenian foods. This involved comparing the results of applying the FSANZ NPSC when the nutritional information from the packaging was supplemented with data from a local food composition table (OPEN)^31, 32^ and the UK Nutrient Databank.

### Analyses

The healthiness of foods was assessed by comparing the mean levels of energy, protein, carbohydrate, total sugars, fat, saturated fat, fibre and sodium of foods carrying health-related claims against foods that do not carry claims. The mean levels per 100 g were chosen, as portion sizes were not always provided by the manufacturer. As the data were not normally distributed, a Mann–Whitney two sample *t*-test was used to determine whether the differences in nutrient levels were statistically significant. All analyses were performed in STATA v11.2.^33^

As some food groups may be more likely to carry claims than others, it was thought that there might also be differences in average nutrient levels between food groups and that any differences between the average levels of nutrients between foods that carry claims and foods that do not may be confounded by the food group. Therefore, a regression analysis was performed that adjusted for food category using the Eatwell Guide food categories. Initially, Kruskal–Wallis tests were conducted to establish associations between food category and (in turn) the presence of health claims and nutritional values. Regression analyses adjusted for food category were then conducted to determine whether there were any statistically significant differences between the mean levels of nutrients.

The FSANZ NPSC was applied to the foods using syntax files, which were checked for consistency by another researcher. The proportion of foods that pass the model was compared by foods that carry health-related claims against foods that do not, using the standard binomial test for proportions. Regression analyses (adjusted for food category) were conducted in order to estimate the mean levels of nutrients for foods that both carried a health claim and passed the FSANZ NPSC model against foods that did not.

## Results

### Missing data

A total of 2034 foods were collected. The provision of on-pack nutritional information differed between countries (Table 1); 31% of foods sampled in Slovenia did not have any nutritional information compared with 8% of foods in the United Kingdom. Overall, 15% of foods did not have any nutritional information and were not included in the analyses of the nutritional composition. Across the five countries, 22% of foods were missing at least one of the selected nutrients (energy, protein, carbohydrate, total sugars, fat, saturated fat, fibre and sodium) and were therefore only included for the nutrient comparisons where that data were available.

For the purposes of applying the nutrient profile model, where only partial nutritional information was available for a product, the data were supplemented with data from the UK nutrient databank. Pearson's R correlation (*r*) test was conducted to check that the nutritional information recorded from the food packaging was well correlated to the matched food in the UK nutrient databank; any outliers were examined and, where necessary, amended.

Overall, energy, protein, carbohydrate, total sugars, total fat and saturated fat had very strong correlations (data available as Supplementary Information) with *r* ranging from 0.80 to 0.93. In some cases, the correlation was lower for foods from a particular country—for example, the correlation for saturated fat was weaker in Spain (0.65) than the other countries (0.83–0.87). Similarly, total sugar was very strongly correlated in four of the countries (0.75–0.89) but was slightly lower in The Netherlands (0.67). Fibre and sodium had weaker correlations, (0.55 and 0.67, respectively), with bigger country variance (Supplementary Table).

Additional analyses were conducted with the Slovenian foods to test the appropriateness of using the UK Nutrient Databank to supplement food composition data for foods from other (non-UK) countries. There was very high agreement (Cohen's kappa 0.90–0.97) between the FSANZ NPSC classifications produced when using the UK Nutrient Databank to supplement the data and when using a Slovenian data source (data and further details available in Supplementary Materials).

### Types of products sampled

Foods and drinks high in fat and/or sugar accounted for 36% of the foods sampled. Meat, fish, eggs, beans, and other non-diary sources of protein, and miscellaneous foods made up 14% and 15% of the database, respectively. Breads, rice, potatoes, pasta and other starchy foods, as well as composite foods, made up 10% of the database each. The remaining two categories, milk and dairy foods and fruit and vegetables, were the smallest categories and each made-up 8% each of foods in the database. There was little country variation in the type of foods sampled from each country; however, there was a greater proportion of foods high in fat and/or sugar sampled from Slovenia (42%) than the other countries (32–38%) and a smaller proportion of foods categorised as Composite foods from Slovenia.

### Prevalence of nutrition and health claims

More than a quarter of foods carried either a health or a nutrition claim; 22% of foods sampled carried a nutrition claim and 11% of foods sampled carried a health claim. The claim prevalence differed by food group, for example, 21% of milk and dairy foods carried a health claim compared with 3% of composite foods (Table 2).

### Mean levels of nutrients

Tables 2 and 3 demonstrate how the food category is associated with both the presence of health claims and the nutritional quality of foods. For example, the prevalence of health claims varied from 21% (95% CI 15%, 27%) in milk and dairy foods to only 3% (CI 1%, 5%) in composite foods, and the energy content of foods varied from 339 kcal/100 g for bread, cereals and potatoes to 79 kcal/100 g for fruit and vegetables.

The levels of energy, protein, and total sugar, total fat, saturated fat and sodium were significantly lower for foods carrying at least one health claim. There was a large difference in the levels of sodium: for foods without health claims, the average amount was 708 mg/100 g compared with 161 mg/100 g in foods with health claims. Smaller differences were seen in the remaining nutrients—for example, foods carrying health claims had mean levels of 6 g/100 g for protein compared with 7 g/100 g for foods not carrying health claims. A similar pattern was observed for foods carrying nutrition claims.

### Adjusting for food category, differences in the mean level of nutrients between foods that carry claims and foods that do not

As the claim prevalence differs by the food group (Table 2) and there were significant differences between the food groups in terms of the mean nutrient levels (Table 3), it was necessary to adjust for food group when assessing the nutritional quality of foods carrying health-related claims (Table 4a, model 2).

Adjusting for the food group reduced the differences in the mean level of some nutrients. For example, in model 1 (no adjustments), the mean difference for total fat was 3.3 g/100 g lower (*P*<0.01) in foods carrying claims, but in model 2 this difference was reduced to 2.1 g/100 g and was non-significant. In contrast, adjusting for food group increased the difference in the mean levels of sodium, 547 mg/100 g lower compared with 842 mg/100 g lower in model 2. Adjusting for food group had little effect on the levels of saturated fat, fibre and protein.

A similar pattern was observed when adjusting for food groups in regard to foods carrying nutrition claims (Table 4b); however, the differences for the mean level of fat (−4 g/100 g) and sodium (−243 mg/100 g) were larger and statistically significant. Foods carrying at least one nutrition claim also had significantly lower levels of energy (−36 kcal/100 g), protein (−1 g/100 g), total sugars (−3 g/100 g), total fat (−4 g/100 g), and saturated fat (−3 g/100 g), and significantly more fibre (+0.9 g/100 g).

In the final section of Table 4a, the mean levels of nutrients are estimated for foods that carry at least one health claim but restricted to foods that pass the FSANZ NPSC, that is, only observing health claims that would be permitted if the current EU regulations were underpinned with the nutrient profile model currently used to regulate health claims in Australia and New Zealand. Foods that carried a health claim and did not pass the FSANZ NPSC were considered as not carrying a claim. In this scenario, in the food group-adjusted model (model 2), there would be significantly lower levels of energy (−56 kcal/100 g), protein (−2 g/100 g), carbohydrates and total sugars (both −7 g/100 g), total fat and saturated fat (both −3 g/100 g), and sodium (−878 mg/100 g), and significantly more fibre (1 g/100 g).

Using the FSANZ NPSC model to restrict health claims would lead to improvements in the mean levels of most nutrients but not all. Foods carrying health claims have, on average, 29 fewer calories per 100 g than foods that do not carry health claims, but if the FSANZ NPSC was used to restrict claims the difference would be 56 calories. Similarly, with regard to the levels of total sugars, foods carrying health claims have, on average, 3 g/100 g less sugar, whereas after the FSANZ NPSC restriction the mean is 7 g/100 g lower. Total fat and protein was 0.4 g/100 g lower, and saturated fat was 0.5 g/100 g lower in foods that carry health claims than when the FSANZ NPSC was not used to restrict (the use of) claims. There was also a 35 mg/100 g reduction in the mean level of sodium but less of an effect on the levels of fibre.

Forty-three percent of the foods sampled ‘pass' the FSANZ NPSC model (Table 5). The percentage that passed the model was similar in each country, Slovenia had the lowest percentage that passed the model (39%, CI 34%, 44%), 40% (CI 35%, 44%) passed in The Netherlands, 42% (CI 37%, 47%) in Germany, 45% (CI 40%, 50%) passed in Spain, and the United Kingdom had the highest pass percentage (48%, CI 43%, 53%). Overall, 36% of foods that do not carry either a health or a nutrition claim pass the FSANZ NPSC; this was similar across the five countries with the lowest pass percentage seen in The Netherlands (31%, CI 26%, 36%). The third column displays the percentage of foods carrying health claims that pass the FSANZ NPSC. Seventy percent (CI 64%, 76%) of such foods passed the FSANZ NPSC. There was greater country variance observed, with the lowest percentage found in Slovenia (51%, CI 37%, 65%) and the highest in The Netherlands (81%, CI 71%, 92%) and the United Kingdom (80%, CI 67%, 92%). Fewer foods carrying nutrition claims passed the FSANZ NPSC, ranging from 50% (CI 39%, 61%) of foods carrying nutrition claims in Slovenia to 73% (CI 65%, 80%) of such foods in the United Kingdom.

## Discussion

Foods that carry health claims have significantly lower levels of energy (−30 kcal/100 g), protein (−1 g/100 g), total sugars (−3 g/100 g), saturated fat (−2 g/100 g), and sodium (−842 mg/100 g), and significantly more fibre (+1 g/100 g) than foods that do not carry health claims (Table 4a, model 2). Foods that carry nutrition claims follow a similar pattern, with significantly lower levels of energy (−36 kcal/100 g), protein (−1 g/100 g), total sugars (−3 g/100 g), total fat (−4 g/100 g), saturated fat (−3 g/100 g) and significantly more fibre (+1 g/100 g) (Table 4b, model 2). Although the differences in protein, carbohydrates, total sugars, total fat, saturated fat and fibre appear to be modest, even small dietary changes can have large impacts on health outcomes when scaled up to a population level.

Small country differences were observed in the nutrient composition of foods with and without claims. The greatest difference was observed with regard to the proportion of foods that carry a health claim and pass the FSANZ NPSC. Slovenia had the lowest proportion of such foods (51%, CI 37%, 65%), whereas The Netherlands had the highest (81%, CI 71%, 92%); however, these analyses were not powered for cross-country comparisons, and any statistically significant (*P*<0.05) differences between countries may be a chance finding as multiple comparisons have been undertaken.

The EC, through its Evaluation and Fitness Check Roadmap,^24^ is seeking to evaluate whether a nutrient profile model is necessary for the regulation of health and nutrition claims and whether the failure to implement such a model has had any negative or even positive effects. The results presented in this paper may be taken to suggest that concerns over the poor nutritional composition of foods carrying health-related claims in Europe may be unfounded given that foods carrying health-related claims have, on average, a better nutritional composition than foods that do not carry such claims. However, 30% of foods carrying health claims and 39% of foods carrying nutrition claims do not pass the FSANZ NPSC. When the FSANZ NPSC was used to restrict health claims, the mean kcal/100 g and total sugars in g/100 g was halved. Smaller improvements, ranging from 0.4 g/100 g to 0.5 g/100 g, were seen in regard to the mean levels of protein, total fat and saturated fat. A smaller difference was seen in the mean levels of fibre when the FSANZ NPSC was used to restrict health claims (0.2 g/100 g less) and there would be a 35 mg/100 g decrease in the mean level of sodium.

To the best of our knowledge, the nutritional composition of foods carrying health claims and nutrition claims has not previously been measured on a multiple country basis using a random selection of foods across all food categories. Previous prevalence studies have typically focussed either on a small number of food categories,^34^ foods that are commonly consumed,^35^ or were audits of foods that carry health or nutrition claims,^36^ whereas this study examined randomly sampled foods from five countries in which most pre-packaged foods were eligible for inclusion.

Where previous studies have evaluated the nutritional composition of foods carrying claims, they have generally involved a restricted number of food groups^37, 38^ and usually within one country. For example, an earlier study of foods in the United Kingdom^21^ found a comparable prevalence of health claims and nutrition claims (29%, CI 25%, 34%) to the current study and also found that foods carrying claims had a slighter healthier nutritional profile than foods that did not. There have also been a number of similar studies to this conducted in Australia that yielded similar findings. For example, one study found that 31% of foods carrying health claims and 29% of foods carrying nutrition claims did not pass the FSANZ NPSC.^39^

One potential weakness of the current study was that the nutritional information collected was incomplete and therefore had to be supplemented with nutritional composition tables from the United Kingdom so that the FSANZ NPSC model might be applied. Because of time and budget constraints local nutritional composition tables were not used. However, validity assessments were conducted to ensure that the supplemented data were as close as possible to those for the sampled foods, and these data were used only for the application of the FSANZ NPSC and not the comparison of the mean level of nutrients. A further analysis of the validity of supplementing the nutritional information collected from packaging was conducted on the sample of Slovenian foods. This involved comparing the results of applying the FSANZ NPSC when the nutritional information from packaging was supplemented with data from a local food composition table (OPEN)^31, 32^ against the results of applying the FSANZ NPSC results when using the UK Nutrient Databank to supplement the information (results not shown but available as Supplementary Material). In summary, there was high agreement between the results (95% agreement, kappa=95%, s.e.: 0.06). The application of data from a food composition database to complete data missing in nutrition declarations has previously been deemed a useful and an effective approach for nutrient profiling of large data sets of foods.^40^ Also, it may be deemed inappropriate to evaluate European foods using an Australian nutrient profile model, as there may be differences in nutritional needs; however, although the FSANZ NPSC is not a European model, it is based on the UK FSA/Ofcom model, which is used to regulate television advertising of foods to children.^41, 42^ An alternative nutrient profile could have been used, such as the EC's proposed model to regulate health claims; however, this model has not been adopted (or published), and therefore we chose a model that was accessible, currently in use and its formative model, the UK FSA/Ofcom model, has been validated against diets in the United Kingdom^43^ and with a survey of nutritional professionals.^44^

A limitation of the study is the use of parametric tests for the adjusted analyses, as the nutritional data were not normally distributed. We used parametric tests in order to adjust for confounding by food category. Future work should involve larger sample sizes so that non-parametric tests may be used in subsamples stratified by food category.

We hope that the results presented in this paper will help the EC assess the need for nutrient profile models in the regulation of health and nutrition claims. Although the nutritional quality of foods carrying claims has been explored in this paper, it is still unclear what the public health impact of these relatively modest differences is. Future work could focus on modelling how the diet may change as a result of health claims and how this may translate into differences in health outcome—for example, by modelling the impact of health claims scenarios such as the introduction of a nutrient profile model to regulate health and nutrition claims.

## Figures and Tables

**Table 1:** Missing data

	*Germany*	*The Netherlands*	*Spain*	*Slovenia*	*United Kingdom*	*Total*
Number of foods (*n*, %, 95% confidence interval)	399, 20% (18%, 21%)	416, 20% (19%, 22%)	405, 20% (18%, 22%)	416, 20% (19%, 22%)	398, 20% (18%, 21%)	2034, 100%
*Missing data* (n, %)						
* *Nutrients						
Energy	55 (14%)	41 (10%)	62 (15%)	128 (31%)	32 (8%)	318 (16%)
Protein	55 (14%)	44 (11%)	61 (15%)	129 (31%)	34 (9%)	32 (16%)
Carbohydrate	55 (14%)	43 (10%)	61 (15%)	129 (31%)	33 (8%)	321 (16%)
Total sugars	109 (27%)	80 (19%)	125 (31%)	196 (47%)	43 (11%)	553 (27%)
Fat	55 (14%)	42 (10%)	61 (15%)	131 (32%)	33 (8%)	322 (16%)
Saturated fat	110 (28%)	79 (19%)	123 (30%)	193 (46%)	43 (11%)	548 (26%)
Fibre	123 (31%)	103 (25%)	161 (40%)	217 (52%)	49 (12%)	653 (32%)
Sodium	111 (28%)	82 (20%)	126 (31%)	208 (50%)	42 (11%)	569 (28%)
Foods without any nutritional information (*n*, %)	54 (14%)	41 (10%)	57 (14%)	128 (31%)	31 (8%)	311 (15%)
Foods with nutritional information for selected nutrients	273 (69%)	306 (74%)	234 (58%)	194 (47%)	348 (87%)	1355 (67%)
Missing nutritional data (%)	21.10%	24.10%	15.40%	40.00%	9.70%	22.20%

**Table 2:** Health-related claim prevalence (*n*, %, 95% CI)

*Eatwell Guide group*	*Number of foods*	*Health claims*	*Nutrition claims*	*Any claim*
Bread, rice, potatoes, pasta and so on	194, 10% (8%, 11%)	23, 12% (7%, 16%)	53, 27% (21%, 34%)	57, 29% (23%, 36%)
Milk and dairy foods	162, 8% (7%, 9%)	34, 21% (15%, 27%)	64, 40% (32%, 47%)	75, 46% (39%, 54%)
Foods and drinks high in fat and/or sugar	740, 36% (34%, 38%)	66, 9% (7%, 11%)	152, 21% (18%, 24%)	172, 23% (20%, 26%)
Meat, fish, eggs, beans and so on	300, 15% (13%, 16%)	24, 8% (5%, 11%)	46, 15% (11%, 19%)	53, 18% (13%, 22%)
Fruit and vegetables	159, 8% (7%, 9%)	16, 10% (5%, 15%)	46, 29% (22%, 36%)	53, 33% (26%, 41%)
Miscellaneous	279, 14% (12%, 15%)	53, 19% (14%, 24%)	62, 22% (17%, 27%)	86, 31% (25%, 36%)
Composite foods	200, 10% (9%, 11%)	6, 3% (1%, 5%)	28, 14% (9%, 19%)	32, 16% (11%, 21%)
Total	2034, 100%	222, 11% (10%, 12%)	451, 22% (20%, 24%)	528, 26% (24%, 28%)

Abbreviation: CI, confidence interval.

**Table 3:** Mean level of nutrients by food category (Kruskal–Wallis test) and claim type (Mann–Whitney test)

	*Energy (KJ/100 g)*	*Energy (Kcal/100 g)*	*Protein (g/100 g)*	*Carbohydrate (g/100 g)*	*Total sugars (g/100 g)*	*Total fat (g/100 g)*	*Saturated fat (g/100 g)*	*Fibre (g/100 g)*	*Sodium (mg/100 g)*
*Eatwell food group*									
Bread, rice, potatoes, pasta and so on	1418.5	339.0	9.1	60.4	8.4	5.6	1.9	5.2	267.3
Milk and dairy foods	699.7	167.2	9.3	7.5	6.7	10.7	6.0	0.2	273.9
Foods and drinks high in fat and/or sugar	1342.8	320.9	3.6	40.3	24.1	16.2	6.3	1.7	262.5
Meat, fish, eggs, beans and so on	1022.3	244.3	16.6	6.9	1.5	16.3	4.6	1.9	809.7
Fruit and vegetables	330.4	79.0	1.8	11.3	8.9	2.9	0.6	2.3	201.5
Miscellaneous	545.1	130.7	3.8	19.5	11.1	3.5	1.5	2.2	2700.7
Composite foods	713.2	170.5	7.7	16.7	4.0	7.8	2.8	1.5	1021.2
*P*-value	0.00	0.00	0.00	0.00	0.00	0.00	0.00	0.00	0.00
									
*Mean levels of nutrients by claim type*									
Without health claims	1051.4	251.4	7.0	28.2	13.7	12.0	4.7	1.9	707.7
With health claims	851.9	203.6	5.5	25.5	10.1	8.7	2.2	2.7	161.1
*P-*value	0.00	0.00	0.00	0.03	0.02	0.00	0.00	0.68	0.00
Without nutrition claims	1078.9	257.8	7.1	27.7	14.1	12.9	5.1	1.8	689.2
With nutrition claims	877.9	210.1	6.1	28.2	10.9	8.0	2.4	2.7	503.6
*P*-value	0.00	0.00	0.00	0.98	0.84	0.00	0.00	0.07	0.00
No health-related claims	1100.7	263.1	7.3	28.2	14.3	13.1	5.3	1.8	715.3
At least one health-related claim	850.0	203.4	5.8	26.9	10.8	7.9	2.3	2.5	469.3
*P*-value	0.00	0.00	0.00	0.12	0.22	0.00	0.00	0.66	0.00

**Table 4a:** Adjusting for food category, differences in the mean level of nutrients between foods that carry health claims and foods that do not, and foods that carry health claims and pass the NPSC and those that do not

	*Health claims*						*Health claims—only those that pass NPSC*					
	*Model 1*	P*-value*	*CI*	*Model 2*	P*-value*	*CI*	*Model 1*	P*-value*	*CI*	*Model 2*	P*-value*	*CI*
Energy (KJ/100 g)	−199.5	0.00	−312.9, −86.8	121.9	0.02	−224.5, −19.4	−370.1	0.00	−500.0, −241.6	−233.8	0.00	−351.0, −116.7
Energy (Kcal/100 g)	−47.8	0.00	−74.9, −20.7	−29.3	0.01	−53.8, −4.8	−88.8	0.00	−119.6, −57.9	−56.0	0.00	−84.0, −28
Protein (g/100 g)	−1.5	0.01	−2.6,−0.4	−1.2	0.01	−2.1, −0.4	−1.9	0.00	−3.1, −0.6	−1.6	0.00	−2.6, −0.6
Carbohydrate (g/100 g)	−2.7	0.20	−6.8, 1.4	−0.7	0.67	−4.0, 2.5	−9.8	0.00	−14.4, −5.1	−6.7	0.00	−9.9, −2.5
Total sugars (g/100 g)	−3.5	0.02	−6.4, −0.7	−3.1	0.02	−5.6, −0.5	−8.4	0.00	−11.6, −5.2	−7.3	0.00	−10.2, −4.4
Total fat (g/100 g)	−3.3	0.01	−5.7, 1.0	−2.1	0.06	−4.4, 0.1	−4.6	0.00	−7.2, −1.9	−2.5	0.06	−5.1, −0.1
Saturated fat (g/100 g)	−2.5	0.00	−3.5, −1.6	−2.4	0.00	−3.3, −1.4	−3.3	0.00	−4.4, −2.3	−2.9	0.00	−3.9, −1.8
Fibre (g/100 g)	0.7	0.01	0.2, 1.3	0.8	0.00	0.3, 1.3	0.9	0.00	0.4, 1.5	1.0	0.00	0.5, 1.6
Sodium (mg/100 g)	−546.7	−2.12	−1052, −40	−842.4	0.00	−1348.4, −336.5	−594.3	0.04	−1166.1, −29.8	−877.6	0.00	−1443.2, −312.0

Abbreviations: CI, confidence interval; NPSC, Nutrient Profiling Scoring Criterion.

**Table 5:** Food Standards Australia New Zealand Nutrient Profiling Scoring Criterion (FSANZ NPSC), (*n*, %, 95% CI)

*Country*	*Foods that pass the FSANZ NPSC*	*Foods that do not carry any claims that pass the FSANZ NPSC*	*Foods that carry health claims that pass the FSANZ NPSC*	*Foods that carry nutrition claims that pass the FSANZ NPSC*
Germany	169, 42% (37%, 47%)	124, 39% (34%, 45%)	26, 68% (53%, 84%)	38, 54% (42%, 66%)
The Netherlands	161, 40% (35%, 44%)	95, 31% (26%, 36%)	48, 81% (71%, 92%)	40, 55% (43%, 66%)
Spain	182, 45% (40%, 50%)	115, 38% (32%, 43%)	20, 67% (49%, 85%)	61, 66% (56%, 75%)
Slovenia	161, 39% (34%, 44%)	113, 36% (31%, 42%)	26, 51% (37%, 65%)	39, 50% (39%, 61%)
United Kingdom	190, 48% (43%, 53%)	90, 35% (29%, 41%)	36, 80% (67%, 92%)	98, 73% (65%, 80%)
Total	863, 43% (41%, 45%)	537, 36% (34%, 38%)	156, 70% (64%, 76%)	276, 61% (57%, 66%)

Missing data supplemented using UK Nutrient databank.

**Table 4b:** Adjusting for food category, differences in the mean level of nutrients between foods that carry nutrition claims and foods that do not, and foods that carry at least one health or nutrition claim and foods that do not carry any claims

	*Nutrition claims*						*Any claim*					
	*Model 1*	P*-value*	*CI*	*Model 2*	P*-value*	*CI*	*Model 1*	P*-value*	*CI*	*Model 2*	P*-value*	*CI*
Energy (KJ/100 g)	−201.0	0.00	−285.0, −117.0	−149.9	0.00	−225.5, −74.3	−250.7	0.00	−339.9, −170.6	−183.1	0.00	−255.9, −110.4
Energy (Kcal/100 g)	−47.7	0.00	−67.8, −27.6	−35.7	0.00	−53.8, −17.6	−59.6	0.00	−78.8, −40.46	−43.7	0.00	−61.1, −26.3
Protein (g/100 g)	−1.1	0.01	−1.9, −0.26	−0.6	0.05	−1.2, 0.0	−1.5	0.00	−2.3, −0.7	−1.0	0.00	−1.6, −0.4
Carbohydrate (g/100 g)	0.5	0.76	−2.6, 3.5	0.9	0.48	−1.5, 3.3	−1.3	0.37	−4.3, 1.6	−0.1	0.92	−2.5, 2.2
Total sugars (g/100 g)	−3.2	0.00	−5.4, −1.1	−3.0	0.00	−4.9, −1.1	−3.5	0.00	−5.5, −1.4	−3.2	0.00	−5.0, −1.3
Total fat (g/100 g)	−4.9	0.00	−6.6, −3.1	−3.8	0.00	−5.5, −2.2	−5.2	0.00	−6.9, −3.6	−4.1	0.00	−5.7, −2.5
Saturated fat (g/100 g)	−2.7	0.00	−3.5, −2.0	−2.6	0.00	−3.3, −1.9	−3.0	0.00	−3.7, −2.4	−2.9	0.00	−3.6, −2.2
Fibre (g/100 g)	0.9	0.00	0.5, −1.3	0.9	0.00	0.5, 1.3	0.7	0.00	0.3, 1.1	0.7	0.00	0.3, 1.1
Sodium (mg/100 g)	−185.6	0.34	−564.0, 192.8	−243.3	0.21	−620.9, 134.4	−246.0	0.19	−610.8, 118.9	−354.7	0.06	−721.3, 11.9

Abbreviation: CI, confidence interval.
