# Erratum: The nutritional quality of foods carrying health-related claims in Germany, The Netherlands, Spain, Slovenia and the United Kingdom

**DOI:** 10.1038/ejcn.2016.196

**Published:** 2016-09-28

**Authors:** A Kaur, P Scarborough, S Hieke, A Kusar, I Pravst, M Raats, M Rayner

**Keywords:** Nutrition, Epidemiology

**Correction to:** *European Journal of Clinical Nutrition* advance online publication 13 July 2016; doi:10.1038/ejcn.2016.114

**Updated online 28 September 2016:** This article was originally published under a standard licence, but has now been made available under a CC BY 4.0 licence. The PDF and HTML versions of the paper have been modified accordingly.

