# Supplementary material for: The nutritional quality of foods carrying health-related claims in Germany, The Netherlands, Spain, Slovenia and the United Kingdom
Source: Eur J Clin Nutr. 2016 Jul 13;70(12):1388–95. doi: 10.1038/ejcn.2016.114 (PMC5153454; doi:10.1038/ejcn.2016.114)
Supplement: Supplementary Information [file ejcn2016114x1.doc]

**SUPLEMENTARY INFORMATION:** Comparison of two data sources: the UK Nutrient Databank and Slovenian OPEN food composition database, for the supplementation of missing nutritional composition data of Slovenian foods sampled for the CLYMBOL project.

**Background and aims**

A database of pre-packaged foods available in Germany, the Netherlands, Spain, Slovenia, and the UK was used to estimate the prevalence and nutritional profile of foods carrying claims (1). In all, 2,034 foods were randomly sampled and the nutritional information (energy, protein, carbohydrates, total sugars, fat, saturated fat, fibre and sodium) was recorded from the nutrient declarations present on food labels. In our paper titled ‘The nutritional quality of foods carrying health-related claims in Germany, the Netherlands, Spain, Slovenia, and the United Kingdom’ we assessed the nutritional composition of foods carrying health-related claims to those that do not through a comparison of the mean levels per 100g and the application of a nutrient profile model currently used to regulate health claims in Australia and New Zealand (Food Standards Australia New Zealand’s Nutrient Profiling Scoring Criterion, FSANZ NPSC) (2).

In order to apply the FSANZ NPSC data on the following nutrients are required; energy, protein, carbohydrates, total sugars, fat, saturated fat, fibre, sodium and calcium. At the time of data collection, the provision of nutritional data was only mandatory for foods that carried health-related claims (3). Therefore, the incomplete nutritional information data was supplemented with the data of similar products in a food composition table, the UK Nutrient Databank (4).

In order to test the appropriateness of using the UK Nutrient Databank to supplement missing data for non-UK foods we measured the strength of the relationship between the nutritional information recorded from the food label and the equivalent nutritional information for the matched product in the UK Nutrient Databank and the Slovenian Open Platform for Clinical Nutrition (OPEN) database (5-7). We then compared the effect of using the OPEN database on the FSANZ NPSC classifications.

**Data sources**

The OPEN database is a web-based tool primarily designed to support dietary assessments and planning. It contains the food composition data for approximately 800 foods that are commonly consumed in Slovenia (8). The database complies with EuroFIR standards that facilitate access to and exchange of comparable, high quality food composition data for industry, regulators and researchers across Europe (9). The OPEN database has also been used in a number of research projects (8, 10-14).

**Methods and analyses**

Each of the Slovenian foods (n=416) was matched with a similar food in the OPEN database. For each matched product the following nutritional information was recorded (per100g); energy, protein, carbohydrates, total sugars, fat, saturated fat, fibre, sodium and calcium. We then compared the nutritional information recorded from the food label to the corresponding nutritional information of the matched products using Pearson’s R correlation statistic. A comparison with the equivalent nutritional information from the UK Nutrient Databank was also performed.

**Effect of different supplementary data sources on FSANZ NPSC score for Slovenian foods**

In order to test the effect of the use of different supplementary data sources on the FSANZ NPSC we applied the FSANZ NPSC model to the Slovenian foods twice. First using the UK Nutrient Databank to supplement the missing food label data, and then using the OPEN database to supplement the missing food label data. The FSANZ NPSC algorithm was applied to the foods using a STATA (15) do-file which was checked by another researcher. The FSANZ NPSC score was converted into a binary variable (pass/fail). We measured the agreement between the models by examining the percentage agreement and Cohen’s kappa (with the standard error). Cohen’s kappa is the preferred method of measuring agreement as it takes chance agreement into account. All analyses were conducted in STATA 11 SE (15).

**Results**

We were unable to identify a suitable food in the OPEN database for two foods and for 10 foods in the UK Nutrient Databank. Of the foods where a matched food was identified, 196 had full data for nutrients required to apply the FSANZ NPSC (energy, protein, total sugars, saturated fat, sodium, calcium and the fruit and vegetable content).

The correlation between nutritional information recorded from the food labels and the nutritional information of the matched food in the OPEN food composition database is presented below.

**Table S1: Correlations (Pearson’s r) between nutritional information recorded from; food labels (‘Label’), the Slovenian Open Platform for Clinical Nutrition database (‘OPEN’), and the UK Nutrient Databank ‘’UK-DB’**

| **Nutrient** | **Label-OPEN** | | **Label-UK DB** | | **UK DB-OPEN** | |
| --- | --- | --- | --- | --- | --- | --- |
|  | **n** | **r** | **n** | **r** | **n** | **r** |
| Energy (kj) | 287 | 0.92 | 279 | 0.93 | 403 | 0.93 |
| Energy (kcal) | 287 | 0.92 | 279 | 0.92 | 403 | 0.93 |
| Protein | 263 | 0.87 | 278 | 0.87 | 352 | 0.86 |
| Carbohydrate | 274 | 0.86 | 278 | 0.87 | 369 | 0.87 |
| Total sugars | 171 | 0.80 | 211 | 0.75 | 286 | 0.85 |
| Fat | 256 | 0.95 | 276 | 0.96 | 346 | 0.91 |
| Saturated fat | 186 | 0.84 | 214 | 0.83 | 338 | 0.76 |
| Fibre | 155 | 0.81 | 190 | 0.48 | 284 | 0.61 |
| Sodium | 185 | 0.38 | 199 | 0.39 | 365 | 0.80 |

The energy, protein, carbohydrate, sugars, fat, and saturated fat and fibre recorded from the food label were strongly correlated with the matched food in the OPEN database with r ranging from 0.80 to 0.95. Whilst a lower correlation was observed for sodium content (0.38).

The correlations between the food label data and the OPEN data were very similar to those between the food label data and the UK Nutrient Databank. The correlations with the UK Nutrient Databank were equal or stronger than the OPEN data for energy, protein, carbohydrate, fat, and sodium. Whilst the correlation for fibre was lower (0.48).

The correlations between the data for foods matched in the OPEN database and the UK Nutrient Databank were also strong, with r ≥0.8 for energy, protein, carbohydrate, total sugars, fat and sodium. The correlations were weaker for saturated fat (0.76) and fibre (0.61).

**Table S2: FSANZ NPSC agreement when using different data sources to supplement missing food label data for Slovenian products**

| **Supplementary data source** | **Foods that pass the NPSC** | **Foods that do not carry any claims that pass the NPSC** | **Foods that carry health claims that pass the NPSC** | **Foods that carry nutrition claims that pass the NPSC** |
| --- | --- | --- | --- | --- |
| UK Nutrient Databank (n= 413) | 161, 39% (CI 34%, 44%) | 113, 36% (CI 31%, 42%) | 26, 51% (CI 37%, 65%) | 39, 50% (CI 39%, 61%) |
| OPEN database  (n = 312) | 144, 46% (CI 41%, 52%) | 98, 42% (CI 35%, 48%) | 25, 76% (CI 60%, 91%) | 38, 61% (CI 49%, 74%) |
| **Agreement: n, % agreement, Kappa (standard error)** | **296, 95%, 0.90 (0.06)** | **221, 94%, 0.88 (0.07)** | **32, 97%, 0.92 (0.17)** | **61, 98%, 0.97 (0.13)** |

A lower proportion of foods pass the FSANZ NPSC when the UK Nutrient databank is used (39%, CI 34%, 44%) to supplement the data from food labels than when the OPEN database is used (46%, CI 41%, 52%). The difference is greatest when looking at the percentage of foods that carry health claims that pass the FSANZ NPSC where 51% (CI 37%, 65%) pass when using the UK Nutrient Databank compared to 76% (CI 60%, 91%) when the OPEN database is used. The differences observed are not statistically significant.

Using Viera and Garrett’s interpretation of kappa (16) there is ‘almost perfect agreement’ between the FSANZ NPSC classification when the UK Nutrient Databank and when the OPEN database is used to supplement missing data. Overall, 95% of foods are categorised in the same way and the agreement increases when looking at foods that carry claims.

**Summary**

Using the UK Nutrient databank to supplement missing nutritional data enables a greater number of foods to be assessed with the FSANZ NPSC that when the OPEN database is used (413 foods and 312 foods, respectively). There are strong correlations between the nutritional data recorded from the food label and the corresponding data from the OPEN database and the UK Nutrient Databank. Similarly, there is very high agreement between the FSANZ NPSC classifications produced when the two data sources are used.

**References**

1: Hieke s, Kuljanic N, Pravst I, Miklavec K, Kaur A, Brown K, Egan M, Pfeifer K, Gracia A, Rayner M. Prevalence of nutrition and health-related claims and symbols on pre-packaged foods: a five country study in Europe. Nutrients, 8, 137; doi:10.3390/nu8030137.

2: Food Standards Agency Australia New Zealand (FSANZ). *Australia New Zealand Food Standards Code - Standard 1.2.7 - Nutrition, Health and Related Claims*. Available from: http://www.comlaw.gov.au/Series/F2013L00054. [Accessed 25^th^ January 2016].

3: European Commission (2006). *Regulation (EC) No. 1924/2006 of the European Parliament and of the Council of 20 December 2006 on nutrition and health claims made on foods 2006.* Available from: http://eur-lex.europa.eu/LexUriServ/LexUriServ.do?uri=CONSLEG:2006R1924:20100302:EN:PDF [Accessed 25^th^ January 2016].

4: Foods Standards Agency. McCance and Widdowson’s Composition of Foods Integrated Dataset. Available from: http://tna.europarchive.org/20110116113217/http://www.food.gov.uk/science/dietarysurveys/dietsurveys/ [Accessed 25^th^ January 2016].

5. OPEN Platform for clinical nutrition [online]. Ljubljana: Institute Jožef Stefan, 2014. Available from: http://www.opkp.si/ [Accessed 20^th^ March 2016].

6. Seljak BK. Computer-based dietary menu planning. J Food Comp Anal. 2009, 22, 414–20.

7. Korosec M; Golob T; Bertoncelj J; Stibilj V; Seljak BK. The Slovenian food composition database. Food Chem. 2013, 140, 495–499.

8. EuroFIR. Available from: http://www.eurofir.org/?page_id=3. [Accessed 11^th^ April 2016].

9. Gurinovic M; Mileševic J; Novakovic R; Kadvan A; Djekic MI; Satalic Z; Korosec M; Spiroski I; Ranic M; Dupouy E; Oshaug A; Finglas P; Glibetic M. Improving nutrition surveillance and public health research in Central and Eastern Europe/Balkan Countries using the Balkan Food Platform and dietary tools. Food Chem. 2016, 193, 173–180

10. Seljak BK; Stibilj V; Pograjc L; Mis NF; Benedik E. Food composition databases for effective quality nutritional care. Food Chem. 2013, 140, 553–561.

11. Bizjak M; Jenko-Praznikar Z; Seljak BK. Development and validation of an electronic FFQ to assess food intake in the Slovene population. Public Health Nutr. 2014, 17(8), 1729–37.

12. Erzen N; Rayner M; Pravst I. A comparative evaluation of the use of a food composition database and nutrition declarations for nutrient profiling. J Food Nutr Res. 2015, 54, 93–100.

13. Benedik E; Seljak BK; Simcic M; Rogelj I; Bratanic B; Ding EL; Orel R; Fidler NM. Comparison of Paper- and Web-Based Dietary Records: A Pilot Study. Ann Nutr Metab. 2014, 64,156–166.

14. Benedik E; Seljak BK; Hribar M; Rogelj I; Bratanic B; Orel R; Fidler NM. Comparison of a web-based dietary assessment tool with software for the evaluation of dietary records. Zdrav Var. 2015, 54(2), 91–97.

15. StataCorp (2009) STATA version 11. College Station, TX, USA.

16. Viera A, Garrett J (2005) Understanding Interobserver Agreement: The Kappa Statistic. Fam Med. 2005 May;37(5):360-3.
